# Things I wish I knew when implementing an ambulatory antimicrobial stewardship program at an urban health system: lessons learned and future directions

**DOI:** 10.1017/ash.2025.187

**Published:** 2025-05-13

**Authors:** Michael P. Veve, Christen J. Arena, Rachel M. Kenney, Brian M. Church, Steven T. Fried, Anita B. Shallal

**Affiliations:** 1 Department of Pharmacy, Henry Ford Health, Detroit, MI, USA; 2 Department of Pharmacy Practice, Eugene Applebaum College of Pharmacy and Health Sciences, Wayne State University, Detroit, MI, USA; 3 Epic Pharmacy Helios Team, Henry Ford Health, Detroit, MI, USA; 4 Department of Family Medicine, Henry Ford Health, Detroit, MI, USA; 5 Department of Infectious Diseases, Henry Ford Health, Detroit, MI, USA

## Introduction

Although contemporary healthcare has evolved from predominantly hospital-based practice to ambulatory settings, most antimicrobial stewardship program (ASP) efforts remains in the hospital.^1^ While 80-90% of antibiotic prescribing occurs in ambulatory settings, only 7% of surveyed United States organizations have an ambulatory ASP and 18% report a single effective stewardship outcome.^1,2^ Challenges for ambulatory ASPs relate to varied resource allocation, a lack of established practice models, competing ambulatory prescribers priorities, ineffective communication strategies, and lack of incentive to prioritize ASP initiatives.^3,4^

**Figure 1.**
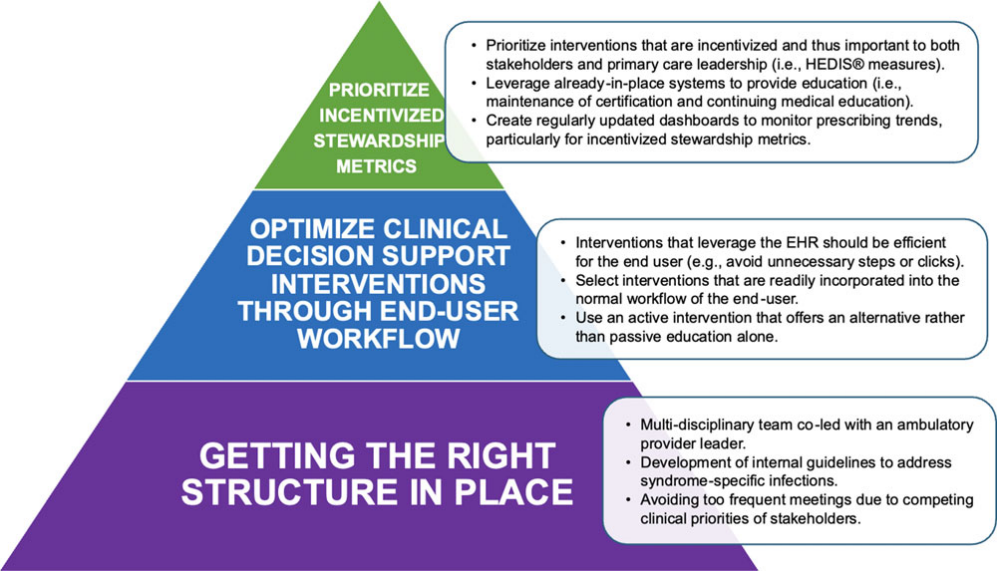
Suggested ambulatory antimicrobial stewardship directions with detailed activities to support program progress.

While “success” in stewardship interventions can be clearly defined based on outcomes, a “failure” is more ambiguous, often felt by the team before outcomes are analyzed. Unfortunately, medical literature favors publications of “success”, leading colleagues to fail in silence or stumble on the same pitfalls as others due to inexperience.^5^ We describe three key strategic directions we wish we knew when establishing an ambulatory ASP at our urban health system across southeast Michigan, and how learning from our previous failures contributed toward eventual progress in program development (Figure 1).

## Getting the right structure in place

A key component to a successful ambulatory ASP is ensuring a structure sustainable for developing and measuring interventions. The three main prerequisites to developing our program included: creation of an engaged multi-disciplinary team, development of internal guidelines, and setting a realistic program pace to account for competing member priorities. Our current ambulatory ASP is co-chaired by an infectious disease (ID) pharmacist, an ID physician, and a family medicine (FM) physician. This leadership approach creates complimentary experience between traditional ASP leaders with a boots-on-ground ambulatory provider who treats patients with common outpatient infections and is fluent in unique workflow challenges foreign to traditionally hospital-based personnel. This leadership triad differs from current recommendations^1^ but allows outpatient providers to be more receptive to ASP interventions or feedback. Our team also includes representation from urgent care, primary care, virtual care, ambulatory pharmacists, information technologists, and payor relations specialists, functioning like an inpatient ASP. The program meets on an every-other-month basis to achieve continued momentum while accounting for competing clinical priorities for providers.

Once the multi-disciplinary team was established, the development of internal syndrome-specific infection guidelines was determined to be a priority and used as a benchmark to identify outlier prescribing. Based on committee feedback, a direct link to the guideline was incorporated into the electronic health record (EHR) to improve accessibility and boosting the guideline click rate. Finally, an ambulatory ASP ideally has designated analyst support to track antibiotic prescribing and guideline adherence.

## Optimize clinical decision support interventions through end-user workflow

Some of the most impactful antimicrobial stewardship interventions involve leveraging the EHR to improve antibiotic decision-making.^6^ These can include development of smart antibiotic order sets or EHR alerts. Generally, these interventions should be active and follow best practices outlined by Smith and colleagues, with emphasis on understanding end-user workflow and their interaction with the EHR enhancement.^6^ Without these considerations, EHR enhancements result in little to no change. Additionally, the ambulatory ASP should gather stakeholder feedback on EHR enhancement design or shadow the end-user to develop a more effective (and utilized) intervention.

Our ambulatory ASP has several examples of EHR enhancements that did not change prescribing due to their passive approach or lack of workflow optimization. We developed indication-based antibiotic order sentence (AOS) prescriptions that contain pre-populated dose and durations for respiratory, urinary, and skin/soft tissue infections in the Emergency Department EHR with the intent to improve antibiotic ordering efficiency and optimization.^7^ After provider education, AOS implementation yielded mild improvements to optimal antibiotic prescribing (8% vs. 23%, *P* < 0.001).^7^ The subsequent implementation of AOS within our primary care network resulted in different results than anticipated, where no statistical differences in optimal prescribing were observed between the pre- and post-AOS groups (25% vs. 29%, *P* = 0.871). As we investigated potential explanations, we hypothesized that provider-saved preference lists that help facilitate antibiotic ordering may have contributed. To address this, the ambulatory ASP voted to remove provider-saved preference lists for fluoroquinolone antibiotics and re-evaluated prescribing data for lower respiratory-tract and urinary-tract infections in ambulatory clinics as a pilot approach. Still, there were no differences in optimal fluoroquinolone prescribing before and after the removal of preference lists (39% vs. 48%, *P =* 0.22).

Future EHR interventions should be active, forward-facing to the prescriber, and persuasive at the time of antibiotic ordering. A recent successful example includes implementation of an outpatient EHR alert for *Clostridioides difficile* infection after national guideline changes. The alert suggested oral vancomycin when prescribers selected metronidazole, which was associated with improved guideline-concordant antibiotic prescribing (72% vs. 91%; *P =* 0.001).^8^

## Prioritize incentivized stewardship metrics

**Figure 2.**
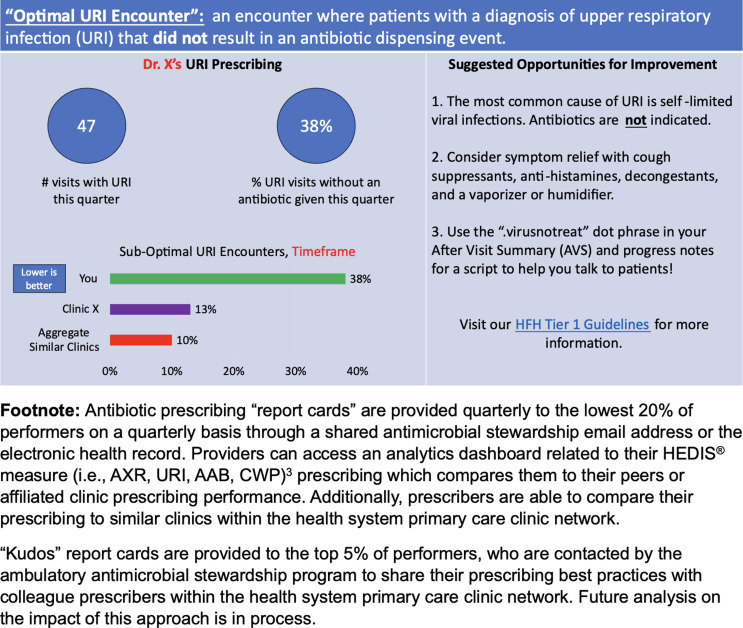
Example provider report card that provides individualized feedback adapted from Healthcare Effectiveness Data and Information Set (HEDIS^®^) measures.

A lack of antibiotic prescribing accountability and performance metrics are significant barriers to ambulatory ASPs. When considering program goals, we recommend leveraging existing pay-for-performance measures, such as Healthcare Effectiveness Data and Information Set (HEDIS^®^) measures, for specific outpatient infections.^3^ Other performance incentives may exist depending on the institution and resources, but ambulatory ASPs should develop relationships with their affiliated payor relations leaders for an individualized approach to HEDIS^®^ measure progress. Our program pivoted to develop HEDIS^®^ measure performance dashboards to monitor prescribing trends. Additionally, we develop and share quarterly individualized HEDIS^®^ measure provider report cards to outlier prescribers with individualized feedback on techniques to improve prescribing (Figure 2).

An initial goal for our ambulatory ASP was to reduce antibiotic durations for common infections. Although resources were allocated to primary care provider education and EHR enhancements, the interventions were ineffective. After these failures, our contemporary practice is to prioritize incentivized performance measures that are tangible to ambulatory provider leadership until additional measures to support ambulatory ASP initiatives (i.e., legislative change) are made.

## Future directions for ambulatory ASPs

Future priorities for our ambulatory ASP include developing successful interventions to promote antibiotic duration of therapy across our health system. Unfortunately, approximately 35% of our health system ambulatory antibiotic prescriptions do not have an indication associated with them. Implementing changes to require antibiotic indications represents a clear next step to optimizing durations of therapy. Additionally, an audit of our outpatient antibiotic prescribing data revealed sinusitis as a top indication that results in an antibiotic prescription despite that most cases of sinusitis are self-limited and not bacterial. Our ambulatory ASP has partnered with an otolaryngologist champion to develop meaningful interventions promoting sinusitis supportive care instead of antibiotic therapy, which has been shown to decrease prescribing in Veterans Affairs settings,^9^ or early otolaryngologist consultation.

Lastly, it is important for the ambulatory ASP to respond to the needs of ambulatory providers when possible. For us, this meant leveraging a locally developed incentive program for continuing medical education so providers could receive maintenance of certification for both direct and indirect reimbursement. An interactive case-based platform was used where antimicrobial stewardship cases focused on optimal testing and treatment of pharyngitis and use of short-course antibiotic therapy. In addition, we responded to provider requests for dot phrases that include “scripts” for avoiding treatment of viral infections and asymptomatic bacteriuria.

The only real mistake is one from which we learn nothing. Trial-and-error proved to be an effective method in development of our ambulatory ASP where published literature is scarce. We encourage other ambulatory ASPs, especially those from better-resourced health systems, to share their own failures and methods so we can be successful together.
